# The Impact of Production Technology on the Quality of Potato Spirit

**DOI:** 10.3390/molecules30224330

**Published:** 2025-11-07

**Authors:** Maria Balcerek, Rafał Mielczarek, Urszula Dziekońska, Katarzyna Pielech-Przybylska, Andrea Patelski

**Affiliations:** Institute of Fermentation Technology and Microbiology, Faculty of Biotechnology and Food Sciences, Lodz University of Technology, Wolczanska 171/173, 90-530 Lodz, Poland; rafalqmielczarek@gmail.com (R.M.); urszula.dziekonska@p.lodz.pl (U.D.); katarzyna.pielech-przybylska@p.lodz.pl (K.P.-P.); andrea.patelski@p.lodz.pl (A.P.)

**Keywords:** potato, starch, yeast strains, fermentation, distillation, ‘okowita’, spirit

## Abstract

Spirit drink, known in Central and Eastern Europe as ‘okowita’ (its official designation is ‘spirit’), is obtained by distilling fermented plant raw materials. Unlike vodka, which is produced from highly purified ethyl alcohol of agricultural origin, ‘okowita’ is characterised by the preservation of the natural aromatic and flavour compounds originating from the raw material and produced during the process of alcoholic fermentation. The study aimed to assess the impact of production technology on the quality of potato spirits. The effects of the methods used for the pretreatment of raw material, starch hydrolysis and fermentation, and yeast strains were examined in relation to the fermentation efficiency and the chemical composition of the distillates. The yeast strains were the key factor determining fermentation efficiency. The SafSpirit and Pinnacle yeast strains provided the highest fermentation yields (85.0–97.7% of the theoretical), while the Ethanol Red strain provided the lowest yield (<83%). No advantage of separate hydrolysis and fermentation (SHF) over simultaneous saccharification and fermentation (SSF) was observed. A characteristic feature of potato distillates was their high isobutyl alcohol content, ranging from 557 to 1437 mg/L of 100% *v*/*v* alcohol, i.e., more than twice that of 3-methyl-1-butanol. Methanol concentrations remained below the limit specified in EU Regulation 2024/1143 (≤1000 g/hL of 100% *v*/*v* alcohol). The results are promising in terms of the potential for the production of craft potato spirit drinks.

## 1. Introduction

During the Middle Ages and the Renaissance, the term ‘aqua vitae’ (and its various dialectical forms), which translates from Latin as ‘water of life’, was widely used to refer to distilled spirits, including those produced by distilling wine, fruits, grains, or potatoes. It is considered a predecessor to vodka but with a more pronounced character and less refinement. Hints of this traditional name can be found in the Scandinavian-language drink Aquavit (Akevitt in Norwegian, Akavit in Danish), which is the national spirit beverage with a strength of 37.5–50% *v*/*v*. The Polish word “okowita” and the Ukrainian word “oкoвитa” (okovita) also originate from this name. The term ‘vodka’ appeared at the end of the 19th century and is a slight variation of the Latin word ‘aqua’, meaning water [1].

In contrast to vodka, which is produced using ethyl alcohol of agricultural origin, namely rectified spirit, ‘okowita’ (spirit) is derived from agricultural distillate and does not undergo full rectification or intensive filtration. Thanks to this, it retains the character and aroma of the raw material from which it was made, thereby ensuring that the finished product retains its original identity [2].

The requirements currently applicable to spirit drinks, known in some European countries as ‘okowita’ or ‘okovita’, are included in Regulation (EU) 2019/787 [3], which governs the production, description, presentation, and labelling of spirit drinks within the European Union countries. In the context of this regulation, potato spirit has been classified as a fruit spirit. Regulation 2024/1143 [4] was introduced in 2024 in response to demands from representatives of the spirits industry, defining potato spirit as a separate type of spirit. The demand for the creation of a distinct category for potato spirits was driven by the necessity to regulate production rules, standardise nomenclature and provide consumers with transparent information on products.

Potatoes, in addition to cereals, are traditional and widely used raw materials for producing spirits intended for food purposes. They can be fermented and distilled to produce high-quality alcoholic beverages, such as vodka and other spirits, which have a distinct sensory profile compared to those made from grains such as rye or corn [5,6]. However, compared to cereals, potatoes contain significantly less starch (from approximately 11 to 22% [7]), which results in a much lower yield of alcohol [8].

Potatoes and sweet potatoes are used in the production of traditional spirits in a number of countries. For instance, they are used in the production of shochu in Japan and lambanog in the Philippines. This further supports their role as important raw materials for food-grade alcohol [9,10,11]. These spirits are valued for their unique flavours and are produced using fermentation and distillation processes analogous to those employed in the production of cereal-based spirits. The utilisation of potatoes not only provides an alternative to cereals but also adds diversity to the range of spirits available for consumption.

Following the European Parliament and the Council of the European Union’s introduction of the official definition of potato spirit [4], a key challenge is to systematically integrate the existing expertise of the distilling industry with current scientific research in this area. This is essential for establishing a conceptual and technological framework that may serve as a reference point for both present and future producers of this legally defined spirit.

When producing spirit distillates from starchy raw materials intended for spirit drinks, it is important to select the appropriate methods of the pretreatment of raw material, hydrolysis and fermentation, as well as the yeast. This ensures correct and efficient fermentation and a desirable qualitative and quantitative composition of volatile compounds in the distillate [12].

The aim of the present study was to evaluate the impact of production technology on the yield and quality of potato spirit. The scope of the work included:

− preparation of potato mashes using pressure-thermal and pressureless starch release methods,
− hydrolysis and fermentation of starch using two strategies, separate hydrolysis and fermentation (SHF) and simultaneous saccharification and fermentation (SSF), and an assessment of the chemical composition of the prepared sweet mashes,
− fermentation of potato mashes using three selected distillery yeast strains,
− assessment of process efficiency,
− determination of the volatile compounds in the potato distillate samples.

## 2. Results and Discussion

### 2.1. Chemical Composition of Raw Material and Sweet Mashes

The potatoes used in the research had a dry matter content of 16.80 ± 0.59%, a starch content of 8.61 ± 0.17% and a protein content of 1.46 ± 0.06%. The starch and protein contents of the tested potatoes were found to be comparable to the values determined by Leonel et al. [13] in the variety Agata (starch 8.89%, protein 1.59%). The chemical composition of potatoes is influenced by a number of factors, including climate, agricultural practice, cultivar, soil composition, and the availability of macro- and microelements [14]. Sawicka and Pszczółkowski [15] indicated that young potatoes harvested at an early stage of physiological maturity exhibit the lowest concentrations of dry matter and total carbohydrates. Despite the comparatively low starch content of the analysed potatoes, this does not preclude their suitability for the production of spirit beverages. In the production of spirits known as ‘okowita’, the sensory attributes of the distillate—namely its flavour and aroma profile—are of greater significance than the high alcohol yield.

**Figure 1:**
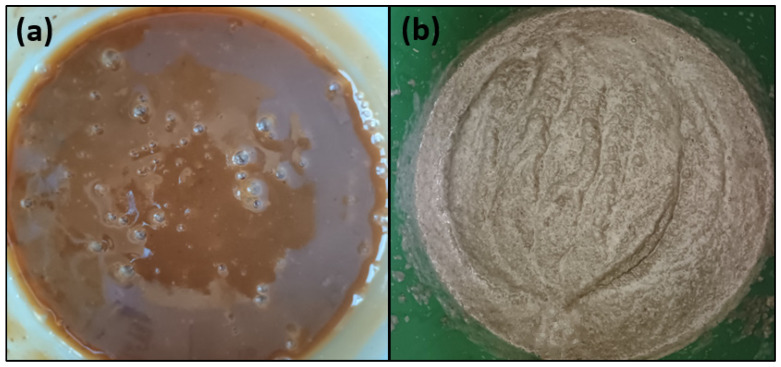
Appearance of potato sweet mashes prepared by the following methods: (**a**) pressure-thermal (PT); (**b**) pressureless starch liberation (PLS).

The tested potatoes were used to prepare distillery mashes. Significant differences in the consistency and colour of the obtained mashes were observed depending on the method used to process the raw material. Sweet mashes prepared using the pressure-thermal (PT) method were in the form of brown, watery liquids containing fine, heterogeneous particles. In contrast, mashes obtained using the pressureless starch liberation (PLS) method were grey-yellow in colour, highly viscous and contained numerous fragments of crushed and cooked potato tubers (Figure 1).

The chemical composition of the mashes is presented in Table 1. Significant differences were observed in the pH value of the potato mashes, depending on the pretreatment method used. The pH level of mashes prepared using the PT method was found to be lower (4.82–5.02) in comparison to mashes prepared using the PLS method (5.91–5.96). Thermal pretreatment of potatoes (especially at temperatures above 100 °C) leads to the formation of mashes with a higher concentration of acidic compounds, resulting from the degradation of potato ingredients, e.g., vitamin C degrades to form oxalic acid and L-threonic acid [16,17].

Refractometric measurements indicated a higher extract content (from 13.40 to 14.50 °Blg) in the mashes prepared using the PLS method. However, these results did not correlate with the total sugar content, which was comparable to or lower than that in the samples prepared using the PT method. The probable reason for this discrepancy is the heterogeneity of the raw material and the different sizes of the tubers, which result in different content of soluble non-starch components, which are part of the extract.

Regarding the impact of the mash preparation method (PT, PLS) and the hydrolysis and fermentation system (SHF, SSF) on the starch saccharification degree and the content of fermentable sugars (i.e., glucose, maltose, maltotriose), no statistically significant differences (*p* > 0.05) were found. Glucose was the predominant sugar, present in all sweet mashes at the highest and comparable concentrations (*p* > 0.05). Moreover, the presence of maltose and maltotriose was noted. Their concentrations were much lower and did not significantly affect the total content of reducing sugars, which was mainly influenced by glucose. The content of total sugars (including those liberated after dextrin hydrolysis) ranged from 79.87 g/L (variant PLS-SHF) to 89.15 g/L (variant PT-SHF). A close correlated with the mash preparation method, as well as the hydrolysis and fermentation system, was not observed. It can be assumed that this variation resulted from the heterogeneity of the raw material. Among the mashes prepared using the PLS method, the one prepared using the separate hydrolysis of starch before fermentation, contained a lower amount of dextrins than that in which hydrolysis and fermentation were carried out simultaneously. This is attributed to the more intense starch hydrolysis during the separate saccharification stage, which is conducted under the conditions that are optimal for amyloglucosidase activity [18]. However, this relationship was not observed for mashes prepared using pressure-thermal treatment of potatoes (Table 1).

### 2.2. Analysis of Fermented Mashes

The prepared sweet mashes were fermented using three strains of distillery yeast: Ethanol-Red, SaftSpirit, and Pinnacle. After completion of fermentation, the mashes were analysed for ethanol, sugars, organic acids, and glycerol concentrations. The results obtained are presented in Table 2.

The main fermentation product was ethanol, with concentrations ranging from 33.03 to 42.85 g/L. Statistical analysis showed no significant differences in ethanol content between the majority of tested mash variants, with the exception of those fermented by the Ethanol Red strain, which exhibited significantly lower values (33.03–37.67 g/L).

As for the content of residual sugars (glucose, maltose, and maltotriose) remaining in the fermented mashes, it was observed that the influence of the yeast strain had a significant influence. More glucose remained in samples fermented with the Ethanol Red strain than in other variants, which proves that this yeast strain utilises glucose less effectively than other strains.

The concentration of maltose was in the range from 0.024 to 0.198 g/L. The highest concentrations were determined in samples prepared using the pressureless starch liberation method (0.083–0.198 g/L), while the lowest were observed in the case when the pressure-thermal treatment of potatoes was applied (0.024–0.090 g/L). Regardless of the hydrolysis and fermentation method and yeast strain used, the highest maltose consumption, compared to the values present in sweet mashes, was noted after fermentation of steamed potato mashes.

Maltotriose concentrations after fermentation ranged from 0.008 to 0.271 g/L. It should be emphasised that these values were closely correlated with the initial levels in the sweet mashes (Table 1), and no significant reduction was observed. A significant decrease in this sugar content compared to the initial value of 0.240 g/L was only observed in the PT-SSF variant.

The dextrin content of the sweet mashes was found to be the result of incomplete starch hydrolysis by amylolytic enzymes. Further dextrin hydrolysis occurred during the fermentation process, resulting in a generation of an additional portion of fermentable sugars. Compared to the initial values, the PT-SHF and PT-SSF variants showed the highest decrease in dextrin content (approximately 13-fold), while the PLS-SHF variant showed the lowest (approximately 2-fold). After completion of potato mashes fermentation, their concentrations ranged from 0.104 to 1.659 g/L.

Comparing the concentrations of fermentable sugars in the sweet and fermented mashes, the degree of their utilisation ranged from 97.0% to 99.0%, indicating almost complete sugar assimilation. These results are higher than those reported by Pielech-Przybylska et al. [19] for barley (between 91.6% and 95.5%). The main reason for the difference in sugar intake may be the initial sugar concentrations in the potato mashes used in the present study and barley mashes tested by the aforementioned authors.

Regarding the presence of by-products in fermented potato mashes, the following compounds were detected: citric acid, succinic acid, acetic acid, formic acid, lactic acid, and glycerol. The lowest citric acid concentrations were observed in the mashes fermented with the Ethanol Red strain (0.653–1.086 g/L), in all samples except the PT-SSF variant. In mashes fermented with other yeast strains, the citric acid concentrations were usually approximately twice as high. Statistically significant differences were demonstrated between the starch hydrolysis and fermentation systems (SHF, SSF) in the mashes prepared from steamed potatoes (Table 2). The SHF variant favoured a higher concentration citric of acid.

In the mashes fermented with the Ethanol Red yeast strain, the concentrations of succinic acid (ranging from 0.297 to 0.947 g/L) and acetic acid (ranging from 0.207 to 3.059 g/L), were found to be statistically significantly higher, especially when the PLS method was employed for the mashes’ preparation. These acids are by-products of anaerobic yeast metabolism [20]. Formic acid was present in the tested mashes at concentrations ranging from 0.290 to 1.516 g/L, demonstrating less dependence on the yeast strain used, while the effect of raw material pretreatment method was observed. In mashes prepared from steamed raw material, the formic acid concentration ranged from 0.290 to 0.723 g/L, while in the samples prepared by the pressureless treatment, its concentrations were significantly higher, ranging from 0.954 to 1.516 mg/L.

Differentiated interactions were also observed for acetic acid in relation to the hydrolysis and fermentation methods, as well as the processing the of raw material. For mashes fermented with SaftSpirit and Pinnacle strains, the acetic acid concentration was lower in the PT-SHF than in the PLS-SHF variant (*p* < 0.05). However, when the SSF strategy was applied, these differences were not observed, and the acetic acid concentrations were similar for mashes prepared using either the pressure-thermal or pressureless potatoes treatment (*p* > 0.05). Steaming ensures the sterilisation of the raw material by eliminating the vegetative forms and spores of undesirable microorganisms. In distilleries, the most common microbial contaminants are lactic acid bacteria (LAB), which ferment carbohydrates to produce lactic and acetic acids. These are the important indicators of the microbiological purity of distillery mashes and the correct course of fermentation [21]. The highest concentrations of acetic and lactic acids were determined in all mashes fermented with the Ethanol Red strain (Table 2). The longer adaptation phase of this yeast during fermentation probably allowed the development of competitive microflora that consumed available nutrients and limited the growth of the yeast population. In contrast, the SaftSpirit and Pinnacle strains rapidly colonised the environment and effectively limited the possibility of bacterial growth through ethanol production.

The concentrations of glycerol, the second most important metabolite of alcoholic fermentation after ethanol, in the potato mashes ranged from 3.359 g/L to 7.271 g/L. Its content was clearly influenced by two technological factors, the most significant of which was the method used to process the raw material. Using the pressureless starch liberation method resulted in the significantly higher glycerol production (4.820–7.271 g/L) than using the pressure-thermal method (3.359–6.779 g/L). The differences caused by the yeast strains were also significant. Mashes fermented with the Ethanol Red yeast strain contained the highest glycerol concentrations (4.457–7.271 g/L). The Pinnacle strain, on the other hand, tended to produce less amounts of this compound (3.359–4.820 g/L). In yeast cells, glycerol serves two key functions. Firstly, it protects yeast cells against osmotic stress—when there is an osmotic potential difference between the environment and the cell interior, glycerol accumulation helps to bridge the current gradient and maintain cellular homeostasis. Moreover, glycerol synthesis plays a significant role in regulating the intracellular redox balance. It is also important to note that the precursor molecule, glycerol-3-phosphate, is also an important substrate for lipid synthesis, which further emphasises the central role of glycerol synthesis in proper cellular function [22]. In the context of the obtained results, consideration should be given to the SSF strategy, which involves limiting the inhibition caused by high glucose concentrations through its gradual release. This is intended to minimise osmotic stress and could consequently result in reduced glycerol production. However, the obtained results showed no statistically significant differences in glycerol concentration between the SSF and SHF strategies, which can be explained by the composition of the sweet mashes (Table 1). The glucose concentration was comparable in all tested variants, meaning the conditions in both variants (SHF and SSF) were similar in terms of inhibitor concentration, which explains the lack of the differences in glycerol production.

### 2.3. Fermentation Efficiency

**Figure 2:**
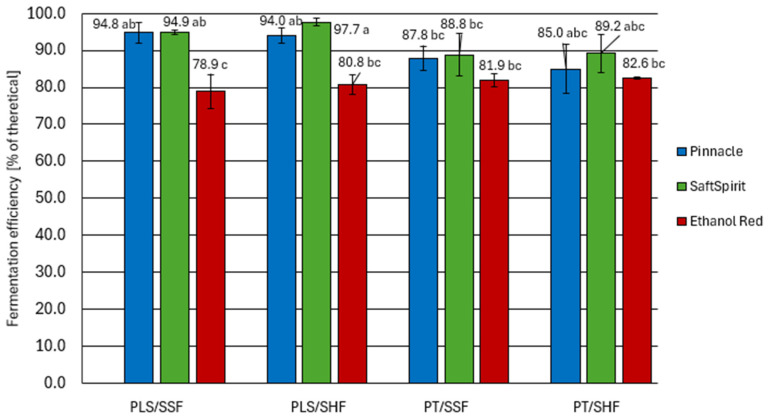
Fermentation efficiency of potato mashes. PT—pressure-thermal starch liberation method; PLS—pressureless starch liberation method; SHF—separate hydrolysis and fermentation; SSF—simultaneous saccharification and fermentation (mean values with different letters differ significantly; multivariate analysis of variance, *p* < 0.05).

The ethanol concentrations in the fermented potato mashes were referred to the calculated theoretical values based on total sugars concentrations, according to the stoichiometric Gay-Lussac equation. Next, the real fermentation efficiency was calculated and expressed as a percentage of the theoretical yield. The results are shown in Figure 2.

Among the yeast strains used to ferment the potato mashes, SaftSpirit and Pinnacle were the most efficient in terms of ethanol production. These strains demonstrated particularly high fermentation efficiency in mashes prepared by the pressureless starch liberation method, achieving yields ranging from 94.0 to 97.7% of the theoretical. These yields exceed those reported for cereal-based mashes described in the literature [19,23,24]. However, it should be noted that the mashes studied by the aforementioned authors contained significantly higher sugar concentrations, which could have had inhibited yeast activity. In contrast, the potato mashes tested by us contained low sugar concentrations, which did not increase the osmotic pressure [25] thereby favouring high yeast fermentation activity.

The fermentation yields obtained for mashes prepared using the pressure-thermal method of starch liberation ranged from 85.0 to 89.2% of the theoretical yield, which is comparable to the values reported in our previous study [26] for cereal mashes. Whilst the steaming of starchy raw materials offers a number of technological advantages in agricultural distilleries, including the effective sterilisation of the feedstock and the release of starch from plant cell structures, this treatment can also induce undesirable chemical transformations. Specifically, reducing sugars present in the raw materials may react with amino acids and peptides during heating, initiating a cascade of Maillard reactions. The resulting Maillard reaction products (MRPs), including furfural and 5-hydroxy- methylfurfural (HMF), have been demonstrated to have an inhibitory effect on *Saccharomyces cerevisiae* [27].

When assessing the impact of starch hydrolysis and fermentation strategies (SHF, SSF), no significant improvement in potato mash fermentation efficiency was observed when separate starch hydrolysis (SHF) was applied under optimal conditions for saccharification enzyme activity. Considering the energy input required for this approach, the extended process time, and the risk of bacterial spores’ germination, the simultaneous saccharification and fermentation approach is undoubtedly recommended for potato processing [28,29].

Fermentation with the Ethanol Red strain yielded lower results (*p* < 0.05) than the other strains, ranging from 78.9% to 82.6%. There was no significant effect of the raw material pretreatment method as well as hydrolysis and fermentation strategy on their activity. It should also be noted that the fermentation with this strain proceeded much slower than with the Pinnacle and SaftSpirit strains. In the samples inoculated with Ethanol Red yeast, a distinct lag phase was observed, lasting approximately 24 h. By this time, the other yeast strains had already reached their maximum growth rate and were undergoing intensive sugar fermentation. The main fermentation stage for Ethanol Red yeast was also significantly prolonged, resulting in a shorter post-fermentation period (with the same total fermentation time for all strains). It can also be assumed that the extended pre-fermentation phase may have led to the development of microorganisms that are undesirable in the spirit production process. These microorganisms were most likely lactic acid bacteria, as they are known to produce lactic acid without concomitant carbon dioxide production, and their presence is a common problem during ethanol production [19].

### 2.4. Volatile Compounds in the Potato Distillates

The quality of agricultural distillates is determined by several factors, including the type and quality of the raw material, its pretreatment method, as well as mashing, fermentation and distillation conditions. During alcoholic fermentation, yeast produces many volatile compounds that influence the final taste and aroma of spirits such as whisky, rum, brandy and others [30].

#### 2.4.1. Carbonyl Compounds

Among the carbonyl compounds present in agricultural distillates, the key compound is acetaldehyde, which is a direct precursor to ethanol biosynthesis. Despite significant variability in its concentrations in tested potato distillates, ranging from 55.28 to 297.17 mg/L alcohol 100% *v*/*v* (Table 3), statistical analysis revealed no significant effect of mash preparation method on its final content. However, a clear trend was observed—for three of the four variants studied, the highest concentrations of this aldehyde were recorded in distillates obtained using the Pinnacle yeast strain. This is in line with the findings of Romano et al. [31], and Li and de Orduña [32], who concluded that the concentration of acetaldehyde varies depending on the yeast species and strain used during the fermentation. Moreover, the application of different raw materials for alcoholic fermentation and varying concentrations of fermenting sugars can affect the concentration of aldehydes in spirit distillates [33]. Acetaldehyde concentrations in tested potato distillates were significantly higher than those reported in the literature for grain raw materials-based distillates. According to the study by Balcerek et al. [26], acetaldehyde concentrations in grain distillates ranged from 3.8 to 16.0 mg/L alcohol 100% *v*/*v*. In turn, Jeleń et al. [34] determined the concentrations of this compound in potato spirits in the range from 8 to 59 mg/L alcohol 100% *v*/*v*. It should be mentioned that the distillates tested by the noted authors had much higher alcohol concentrations than our samples, which were only concentrated to approximately 43% *v*/*v*, resulting in the presence of significant amounts of volatile compounds in the final product. Regulation (EU) 2024/1143 of the European Parliament and of the Council does not currently specify a minimum alcohol concentration for potato distillate used to produce a potato spirit drink (‘okowita’) [4], while according to the Polish Standard [35], the concentration of aldehydes, expressed as acetaldehyde, in agricultural distillates should not exceed 100 mg/L alcohol 100% *v*/*v*.

The organoleptic properties of acetaldehyde depend on its concentration. When present at high levels (above 125 mg/L), it can have a negative impact on the sensory profile of spirits, giving them a pungent and irritating aroma [36]. However, it should be noted that the effect produced by acetaldehyde depends heavily on the matrix in which it is found [37]. The great variability of the sensory thresholds for acetaldehyde, ranging from 0.5 to 100 mg/L [38], illustrates this reactivity. Apart from its effect on the sensory characteristics of spirits, acetaldehyde is a substance classified as ‘possibly carcinogenic to humans’ (Group 2B) by the IARC [39]. This indicates the need to limit its content in alcoholic beverages. According to the literature [40], the low boiling point and high solubility of acetaldehyde in ethanol may allow this compound to be separated during fractional distillation.

Regarding the acetone content (Table 3), a statistically significant effect of the raw material processing method was observed. The use of pressureless starch liberation (PLS) method resulted in 2–5 times higher concentrations (from 8.70 to 21.46 mg/L alcohol 100% *v*/*v*) of this compound in the distillates compared to the pressure-thermal method (2.78–5.13 mg/L alcohol 100% *v*/*v*). The main substrate in acetone synthesis is acetyl-CoA. Two acetyl-CoA molecules condense to form one acetoacetyl-CoA molecule (catalysed by acetoacetyl-CoA synthase), from which acetoacetate is formed in a reaction catalysed by acetoacetyl-CoA transferase. In the final step, acetoacetate is decarboxylated to form acetone by acetoacetate decarboxylase [41]. The effect of acetone on the smell of spirits is described as ‘chemical’ or ‘solvent-like’. Studies on the sensory profile of distilled soju found that the presence of an ‘acetone smell’ was among the least preferred aroma attributes and negatively affected overall consumer acceptance [42]. It should be noted that the acetone concentrations in the tested potato distillates were at least 10 times lower than its detection threshold (200 mg/L) [43], indicating no effect on the quality of the distillates. Diacetyl is formed during alcoholic fermentation through the spontaneous decarboxylation of α-acetolactate, which is a precursor in the synthesis of valine. The yeast *S. cerevisiae* has been shown to efficiently reduce diacetyl to acetoin and subsequently to 2,3-butanediol. This rapid metabolism is particularly responsible for the small amounts of diacetyl produced during the yeast growth phase. However, the formation of acetoin primarily depends on the NAD+/NADH ratio and the intracellular pyruvic acid concentration [44]. In potato distillates, diacetyl concentrations ranged from 4.07 to 34.79 mg/L alcohol 100% *v*/*v*, whereas acetoin contents were between 2.10 and 23.83 mg/L alcohol 100% *v*/*v*. No clear correlations were found between the content of these compounds and the raw material pretreatment method (PT, PLS), hydrolysis and fermentation (SSF, SHF), or yeast strains. Furthermore, no acetoin was detected in the distillates from mash samples prepared by steaming and fermented with the Ethanol Red yeast strain (Table 3). Hexanal was not detected in the distillates obtained from steamed potatoes. However, the samples obtained from the raw material processed by the pressureless starch liberation method exhibited concentrations ranging from 0.63 to 1.6 mg/L alcohol 100% *v*/*v*. Diacetyl and acetoin are important fermentation by-products that significantly shape the aroma profile of alcoholic beverages. Diacetyl is one of the key odour compounds responsible for the typical sensory descriptors attributed to freshly distilled Cognac spirits [45]. The sensory impact of these compounds depends on their concentration and the beverage matrix. Diacetyl is renowned for its strong ‘buttery’ or ‘butterscotch’ aroma, which is detectable at very low concentrations (threshold: 0.1–0.2 mg/L in beer, 1–2 mg/L in wine). At low levels, it can add pleasant buttery notes; however, above the threshold level, it is widely considered an off-flavour, giving beer or wine a spoiled or artificial taste. Higher concentration of ethanol in a solution can decrease the sensory perception of diacetyl, potentially increasing its threshold, as demonstrated in studies of ethanol-water solutions [46]. Acetoin has a much higher sensory threshold (150 mg/L in wine, 8.2 mg/L in beer) and a milder, creamy aroma. Its direct impact on aroma is less pronounced, but it can contribute to the overall mouthfeel and subtle creamy notes. [47]. While low levels of diacetyl can add complexity, excessive amounts are undesirable. Due to its higher threshold and milder aroma, acetoin is less likely to cause undesirable experiences, but it can still influence the perception of smoothness [48].

With regard to the content of furfural, which is mainly produced during the thermal treatment of starchy raw materials [49], its occurrence in potato distillates was observed in a wide range from 3.94 to 15.38 mg/L alcohol 100% *v*/*v*. The observed concentrations are significantly lower than those determined by Balcerek et al. [26] in cereal distillates (ranging from 3.1 to 70.1 mg/L alcohol 100% *v*/*v*). It should further be noted that the highest concentration of furfural was found in potato distillate from the raw material after pressureless treatment and fermentation with the Ethanol Red yeast strain. This suggest that there are other routes to its formation. Furfural can also be formed during the process of distillation in which Maillard reactions occur [50]. The odour thresholds of furfural determined in spirits with an alcoholic strength of 40% *v*/*v* is 122 µg/L [51]. The levels of volatile furanic compounds, including 5-methylfurfural, and furfural are affected by the distillation method. Double distillation results in higher concentrations of these substances [52]. Furfural is a key volatile compound that influences the sensory quality of spirits such as brandy, whisky, rum, and baijiu. Its smell has been described as roasted, sweet, and woody [53].

Decanal was found in potato distillates at concentrations ranging from 4.55 to 34.45 mg/L alcohol 100% *v*/*v*. Its concentration was the highest in mash samples fermented using the Ethanol Red yeast strain. The exception was the PLS-SHF combination, for which the decanal content in the distillate was lower than in samples fermented with Pinnacle and SaftSpirit yeast. These differences were not related to the composition of the mashes or the method of their preparation. Zhao et al. [54] observed during a study of the fermentation of Douchi, a traditional fermented soybean product, that decanal production was positively correlated with the abundance of certain yeast species, particularly in the later stages of fermentation. This suggests that yeast succession and diversity play a role in decanal formation during complex fermentations. Research indicates that the presence of decanal is positively correlated with the sensory quality and aroma complexity of spirits such as brandy. Although decanal is not usually the dominant aroma compound, its presence is associated with an enhanced aroma complexity and quality of spirits. It is one of several volatiles that collectively shape the sensory profile, especially in aged products. Alongside other volatiles such as furfural, decanal contributes to the evolving and desirable aroma profile of aged spirits [55]. The concentration of acetaldehyde diethyl acetal ranged from 22.47 to 96.84 mg/L alcohol 100% *v*/*v*. A statistically significant relationship was observed between the content of this acetal and the acetaldehyde concentration. Samples with lower acetaldehyde content were found to have a higher level of the corresponding diethyl acetal, indicating a dynamic balance between these compounds. According to the Regulation (EU) 2022/1303, the acetaldehyde limit in ethyl alcohol of agricultural origin includes the combined content of ethanol and 1,1-diethoxyethane (acetaldehyde diethyl acetal) [56]. Acetaldehyde diethyl acetal has an odour threshold of 69 µg/L and contributes to the aroma and flavour profile of spirits, often imparting fruity or floral notes. It is considered important for the sensory quality and balance of distilled beverages [57]. The concentration of acetaldehyde diethyl acetal may vary significantly depending on the type of spirit, the raw materials used, the fermentation and distillation processes, and the storage conditions. For instance, plum distillates contain this compound at concentrations ranging from 97.5 to 363.2 mg/L alcohol 100% *v*/*v*, whereas rye distillates contain significantly lower amounts ranging from 0.75 to 5.2 mg/L alcohol 100% *v*/*v*. Moreover, an increase in its concentration was observed during the storage of spirits [58]. In some spirits, acetal is linked to the coordination and harmony of the fragrance, while in others, it can contribute to pungency or off-flavours if present in high concentrations. This depends on the type of alcoholic beverage and the proportions of the individual aroma components [59].

#### 2.4.2. Esters

Esters play a crucial role in the desirable aroma and flavour of spirits, especially when present in balanced concentrations. They enhance desirable notes such as fruit and freshness, as well as overall aromatic complexity. However, excessive levels can introduce negative, solvent-like qualities [60].

The content of esters in the potato distillates tested showed significant variability. Ethyl acetate was the dominant ester, reaching the highest concentrations in the range of 60.47 to 186.69 mg/L alcohol 100% *v*/*v* (Table 4). For comparison, the average concentrations of this compound in rye and potato distillates were 199 and 350 mg/L alcohol 100% *v*/*v*, respectively, according to the study by Jeleń et al. [34]. The lower values observed in the potato distillates tested in our study may indicate a lower potential for esters formation by the yeast strains used. While this is a desirable feature in the production of ethyl alcohol with a low content of volatile compounds, it may limit the production of alcoholic beverages where taste and smell are key factors. It should also be noted that an increased ester content, including ethyl acetate, may indicate the development of microbiological infections in the production of agricultural distillate [61].

The average concentration of ethyl acetate (96 mg/L alcohol 100% *v*/*v*) was determined in the distillates obtained after using steaming for potatoes pretreatment and was significantly lower (*p* < 0.05) compared to the samples obtained when the pressureless method for potatoes pretreatment was applied (134 mg/L alcohol 100% *v*/*v*). Regarding samples from pressure-thermal pretreatment, significantly lower concentrations of ethyl acetate were found in the distillates produced according to the SHF system (60.47–81.69 mg/L alcohol 100% *v*/*v*) than in those produced using the SSF system (113.36–134.18 mg/L alcohol 100% *v*/*v*). This relationship was not observed in spirits obtained from mashes produced using the pressureless starch liberation method.

Other esters were present at much lower, trace levels, not exceeding several milligrams per litre of absolute alcohol, but they were probably still crucial for shaping the aroma of distillates. 3-methylbutyl acetate (isoamyl acetate) is one of the most important fruit esters produced by *S. cerevisiae* yeast from amyl alcohol [62]. The potato distillates contained this compound at the concentrations ranging from 0.51 to 8.00 mg/L alcohol 100% *v*/*v*. Its significantly lower concentrations were recorded in the distillates obtained after fermentation with the Ethanol Red strain (from 0 to 0.56 mg/L alcohol 100% *v*/*v*). In the case of distillates obtained after fermentation by the Pinnacle and Saft Spirit yeast strains, higher concentrations of 3-methylbutyl acetate were observed in the PLS variant (2.07–8.00 mg/L alcohol 100% *v*/*v*) compared to the PT variant (1.36–3.24 mg/L alcohol 100% *v*/*v*). Fatty acid esters, such as ethyl hexanoate, ethyl octanoate, ethyl nonanoate and ethyl decanoate, occurred within the following concentration ranges: 0.62–2.05 mg/L alcohol 100% *v*/*v*, 3.51–10.86 mg/L alcohol 100% *v*/*v*, 1.07–5.10 mg/L alcohol 100% *v*/*v* and 3.00–12.42 mg/L alcohol 100% *v*/*v*, respectively. The concentrations of ethyl hexanoate did not change significantly depending on the technological variant used. In contrast, the content of ethyl octanoate was significantly increased when the PLS method was used to prepare potato mashes. Concentrations of ethyl nonanoate differed significantly between variants, but there was no clear trend. In four distillate samples, the presence of this ester was not detected when SaftSpirit and Pinnacle yeast strains were used for fermentation, while in other combinations they achieved its highest concentrations. In the case of ethyl decanoate, a clear tendency for the Pinnacle yeast strain to produce this compound intensively was observed (the concentration ranges: 6.08–12.42 mg/L alcohol 100% *v*/*v*) compared to Ethanol Red strains (3.00–5.67 mg/L alcohol 100% *v*/*v*) and SaftSpirit (4.74–8.33 mg/L alcohol 100% *v*/*v*).

The presence of ethyl formate and ethyl propionate was detected in distillates obtained from potatoes subjected to pressure-thermal pretreatment at concentrations ranging from 1.47 to 6.18 mg/L alcohol 100% *v*/*v* and from 0.32 to 2.16 mg/L alcohol 100% *v*/*v*, respectively. In contrast, the majority of distillates obtained from potatoes treated by the PLS method did not contain these compounds (Table 3). It can be assumed that the elevated temperature and controlled moisture levels during pressure-thermal treatment of starchy raw materials promote esterification reactions. Lie et al. [63] observed that combining citric acid treatment with heat-moisture treatment in wheat starch significantly increased esterification, as confirmed by FT-IR.

Literature data indicate that spirits such as whisky, brandy, rum, and others typically contain ethyl acetate as the predominant ester, present in concentrations ranging from several tens to hundreds of milligrams per litre. This ester has a relatively high odour threshold of approximately 7.5 mg/L. At low concentrations, it contributes to pleasant, light, fruity notes, whereas at higher levels, it may produce an undesirable, solvent-like odour [64].

The most important sensory-active esters commonly identified in distilled spirits, include ethyl hexanoate (odour threshold: 55 µg/L), ethyl octanoate (odour threshold: 12.9 µg/L), ethyl decanoate (odour threshold: 1120 µg/L), isoamyl acetate (odour threshold: 93.9 µg/L), ethyl butanoate (odour threshold: 81.5 µg/L), and 2-phenylethyl acetate (odour threshold: 108 µg/L). These esters are known to contribute significantly to fruity, floral, and sweet aromas. For example, medium-chain ethyl esters (such as ethyl hexanoate, octanoate and decanoate) impart apple-, melon- or floral-like characters, whereas acetate esters (such as isoamyl acetate and 2-phenylethyl acetate) provide banana-, pear- or rose-like aromas. Analyses of rums and fruit-based distilled spirits repeatedly identify these compounds as key odorants and confirm their relevance to the aroma profile [51,64,65,66]. In whisky, esters such as ethyl hexanoate, ethyl octanoate, and ethyl (S)-2-methylbutanoate are key contributors to the sensory characteristics, although their impact can be partially masked by woody compounds derived from maturation [67,68]. In rum, ethyl butanoate and ethyl (S)-2-methylbutanoate are among the most aroma-active esters, contributing to characteristic sweet-fruity notes [66].

Overall, it is not the absolute concentrations but rather the balance and interaction of esters with other volatile compounds (e.g., higher alcohols and acids) that ultimately shape the final aromatic profile of spirit beverages [64,67].

#### 2.4.3. Methanol and Higher Alcohols

Methanol occurs naturally in many alcoholic beverages as a result of pectin degradation during fermentation [69]. Although methanol does not directly influence the flavour of spirits, it is strictly regulated due to its high toxicity [70].

Methanol is a limited component in potato spirits. The maximum permissible content of methanol in such a product is 1000 g/hL 100% alcohol *v*/*v*, i.e., 10 g/L of 100% alcohol by volume [4]. The methanol concentrations in the potato distillates ranged from 699.99 to 1530.95 mg/L alcohol 100% *v*/*v*. The statistical analysis for this compound confirmed the effect of all the applied parameters, i.e., method of raw material processing (PT, PLS), system of hydrolysis and fermentation system (SSF, SHF), and yeast strains.

The highest methyl alcohol content was found in the distillates from mashes prepared using the pressure-thermal method and ranged from 1076.89 to 1530.95 mg/L alcohol 100% *v*/*v* (Table 5). An increase in the content of this compound was also observed in the spirits from steamed potatoes when the SSF technique was used. In turn, the distillates from mashes prepared using the pressureless starch liberation method, contained this compound at the levels ranging from 699.99 to 902.98 mg/L alcohol 100% *v*/*v*. The observed relationships between methanol concentrations and the method of mash preparation are consistent with the results obtained by Pielech-Przybylska et al. [19]. These authors observed higher methanol concentrations in spirits obtained after fermentation of cereal grain mashes prepared by the pressure-thermal method (approx. 150 °C) than with the pressureless method (90 °C). Moreover, it was observed that highest methanol concentrations were determined in the samples obtained from mashes fermented using the Ethanol Red yeast strain (Table 5).

The high methanol concentration compared to rye mashes is consistent with the data described by Jeleń et al. [34]. The authors also noted that, among the agricultural distillates studied, the highest average methanol concentration was determined in potato distillates and amounted to 1173 mg/L alcohol 100% *v*/*v*. However, it should be noted that all of the potato distillates obtained in our study contained significantly lower concentrations of methanol than the limit specified in the EU Regulation 2024/1143 [4].

From a quantitative point of view, a significant group of fermentation by-products are higher aliphatic alcohols. These are primarily represented by 1-propanol, 2-methyl-1-propanol, 1-butanol, 3-methyl-1-butanol, and amyl alcohol, along with its isomers, i.e., 2-methyl-1-butanol and 3-methyl-1-butanol. These compounds play an important role in creating the flavour characteristics of spirits, including whisky and others [71].

Among the higher aliphatic alcohols present in the potato distillates studied, 1-propanol and 1-butanol were present at relatively low levels. The concentration of 1-propanol ranged from 198.13 to 1183.14 mg/L alcohol 100% *v*/*v*, while 1-butanol was present at even lower levels ranging from 4.03 to 27.03 mg/L alcohol 100% *v*/*v* (Table 5). Despite the difference in absolute values, both compounds showed a similar trend, with the highest concentrations almost always occurring in distillates obtained after fermentation with the Pinnacle yeast strain. These concentration ranges also coincided with the results of the study by Jeleń et al. [34], in which the average concentrations of 1-propanol and 1-butanol in potato distillates were determined to be 865 mg/L alcohol 100% *v*/*v* and 55 mg/L alcohol 100% *v*/*v*, respectively. Regarding 1-propanol, the potato distillates obtained in our study were characterised by a lower concentration than the grain distillates described in the study by Balcerek et al. [26], in which the maximum concentration exceeded 2000 mg/L alcohol 100% *v*/*v*.

Branched-chain higher alcohols, i.e., 2-methyl-1-propanol (isobutanol), 3-methyl-1-butanol (isopentanol), and 2-methyl-1-butanol (active amyl alcohol), constituted the majority of the identified higher alcohols. Among them, isobutanol was present at the highest levels, ranging from 557.54 to 1436.99 mg/L alcohol 100% *v*/*v*. The highest concentrations were found in the distillates obtained using the PLS method and the SaftSpirit and Pinnacle yeast strains (ranging from 1255.6 to 1437 g/L alcohol 100% *v*/*v*), while the lowest concentrations were found in case of the Ethanol Red strain (from 557.54 to 829.62 g/L alcohol 100% *v*/*v*). The concentrations of 3-methyl-1-butanol and 2-methyl-1-butanol were in the ranges of 316.58–639.71 mg/L alcohol 100% *v*/*v* and 166.20–415.80 mg/L alcohol 100% *v*/*v*, respectively. The levels of isobutanol and isoamyl alcohol mainly depend on the amino acid content, particularly valine and leucine, in the fermentation medium, and on yeast metabolism [72].

Compared to literature data on grain distillates [19,26], significant differences in the profile of higher alcohols were observed. In all the cited studies, the fusel alcohol with the highest concentration was 3-methyl-1-butanol, with an average content approximately 1.2–1.3 times higher than that of 2-methyl-1-propanol. However, in the tested potato distillates, a different relationship was observed—the dominant alcohol was 2-methyl-1-propanol, with a concentration more than twice that of 3-methyl-1-butanol. These results indicate that the predominance of isobutanol over isopentanol could be a distinguishing feature of potato distillates, providing a basis for identifying the raw material origin of the distillates.

The effects of higher alcohols on the aroma and taste of alcoholic beverages are extremely dependent on the type of alcohol and its sensory properties. Carmeleyre et al. [73] conducted a descriptive analysis and observed that the addition of isobutanol and isoamyl alcohol (88 and 43 mg/L, respectively) to simple aroma mixtures of fruity esters and acetates significantly decreased in the intensity of fresh and fruity attributes while increasing the spirit-like aroma intensity of the mixture.

These compounds play a key role in shaping the flavour of whisky and other distilled beverages. Malt Scotch whiskies, in particular, are rich in higher alcohols, often exceeding concentrations of 2 g/L [74]. In turn, the maximum allowable concentration of these compounds in rye distillates used in the production of a spirit known as Starka is 5 g/L of absolute alcohol [35].

According to Rodríguez Madrera et al. [75], total concentrations of higher alcohols exceeding 350 g/hL of absolute alcohol result in a strong, pungent, and fusel aroma profile in spirits. However, when present at moderate levels, these compounds enhance the desirable aromatic complexity of spirits.

Aylott and MacKenzie [76] investigated the authenticity of Scotch whisky. They found that the concentrations of 2- and 3-methylbutanol in grain Scotch whisky were relatively low compared with n-propanol and isobutanol. In contrast, malt Scotch whiskies contained substantially higher levels of these higher alcohols—on average, 190 g per 100 L of absolute alcohol, compared to only 30 g per 100 L in grain Scotch whiskies. Moreover, the quality of whisky can be evaluated by calculating the amyl alcohols–isobutanol and isobutanol–1-propanol ratios, which should be greater than 1 [77].

Higher alcohols, which are present in alcoholic beverages including spirits, differ in their sensory descriptors, and odour thresholds. Among these alcohols, 1-propanol, 2-methyl-1-propanol, 3-methyl-1-butanol, and 2-methyl-1-butanol are associated with fusel, solvent-like, and pungent aromas, with odour thresholds ranging from 40 to 56 mg/L. Although such alcohols can contribute to the body and depth of flavour at low concentrations, excessive levels are associated with sharp, harsh notes typical of low-quality distillates [78]. In turn, 1-butanol, 1-pentanol, and 1-hexanol exhibit alcoholic, fruity, or green odour notes, with moderate odour thresholds ranging from 1 to 5 mg/L. Their presence at appropriate concentrations enhances the pleasant, fresh, and fruity character of spirits. These alcohols often originate from raw plant materials or lipid metabolism pathways [51]. In spirits such as brandy, rum and whiskey, a higher content of fusel alcohols, in particular n-propanol, n-butanol and isobutanol, is crucial for the sweetness and taste, as they are the basic ingredients of some esters, resulting in unique flavours [43]. However, it should be noted that the final sensory perception of a spirit depends on the quantitative balance between volatile compounds rather than on their individual concentrations alone [53].

#### 2.4.4. Acidity

One of the key parameters used in distilleries to assess the quality of agricultural distillates is total acidity, expressed in grams of acetic acid per litre of 100% *v*/*v* ethyl alcohol, even though EU regulation [3] does not specify any limits for acidity in agricultural distillates. According to the recommendation of the national standard [38], the acidity of potato-based agricultural distillates, should not exceed 80 mg of acetic acid per litre of alcohol 100% *v*/*v*. Distillates obtained using the Ethanol Red yeast strain for fermentation were found to have acidity levels ranging from 0.068 to 0.125 g acetic acid/L alcohol 100% *v*/*v*. Maximum values, exceeding the limit recommended in the above-mentioned standard, were recorded for the PT-SSF and PLS-SSF variants.

**Figure 3:**
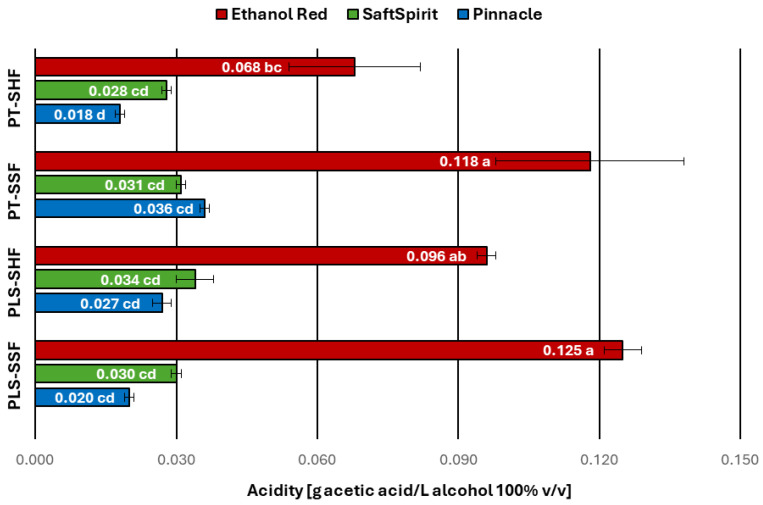
Acidity of potato distillates. PT—pressure-thermal method of starch liberation; PLS—pressureless method of starch liberation; SHF—separate hydrolysis and fermentation; SSF—simultaneous saccharification and fermentation (mean values with different letters indicate significant differences; multivariate analysis of variance, *p* < 0.05).

Of the distillates tested, one sample obtained using the Ethanol Red strain (PT-SHF variant) and all samples produced with the Pinnacle and SaftSpirit strains exhibited acidity levels below the permissible limit. The distillates obtained with the Pinnacle and SaftSpirit strains showed acetic acid concentrations ranging from 0.018 to 0.036 g/L alcohol 100% *v*/*v* and from 0.028 to 0.034 g/L alcohol 100% *v*/*v*, respectively, without statistically significant differences between these two strains (Figure 3).

The odour threshold of acetic acid is 160 mg/L alcohol 40% *v*/*v* [51] and its organoleptic features are described as vinegary and acidic. It is mainly discarded with the tail fraction during distillation [53]. Regarding the potato distillates obtained in this study, it should be noted that further strengthening by distillation and separation of the tail fraction would reduce their acidity, thus enabling the required quality to be achieved.

## 3. Materials and Methods

### 3.1. Raw Material and Auxiliary Materials

The raw materials used to produce the spirit were potatoes (variety Denar), which were obtained from the local market.

The following enzyme preparations (Novonesis, Denmark) were used for the mashing process:

− LpHera—a preparation with α-amylase (EC 3.2.1.1) activity, applied at a dose of 0.50 mL/kg of starch,
− Saczyme—a preparation with amyloglucosidase (EC 3.2.1.3) activity, applied at a dose of 0.72 mL/kg of starch [79].

The following yeast strains of the species *Saccharomyces cerevisiae* were used for alcoholic fermentation of the prepared mashes (each one at a dose of 0.5 g/L of mash):

− Ethanol Red^®^ (Fermentis Division of S.I. Lesaffre, Marcq-en-Baroeul Cedex, France),
− SaftSpirit^TM^ HG-1 (Fermentis Division of S.I. Lesaffre, Marcq-en-Baroeul Cedex, France),
− Pinnacle Distillers Yeast (S) (AB Biotek, Peterborough, UK).

### 3.2. Preparation and Fermentation of Sweet Mashes

Sweet mashes were prepared using two methods of raw material pretreatment and hydrolysis and fermentation, giving a total of four combinations (Table 6).

The initial step, irrespective of the variant, was the washing of the raw material. The potatoes were cleansed of any residual soil by washing them under an intense stream of cold tap water. In the case of mashes prepared by a pressure-thermal pretreatment, the potatoes were then introduced into Henze’s steamer. The process was initialised with a preliminary steaming phase, during which water vapour with a pressure of 1 atmosphere and a temperature of 120 °C was passed through the raw material. The presence of starch in the condensate stream was detected using a coloured reaction with the Lugol’s iodine solution (1% *w*/*v*). This indicate that the valves should be closed, and the vapour pressure increased to 4 atmospheres (152 °C). The main steaming stage lasted for 40 min, after which the content of the Henze steamer was transferred into a mash tun equipped with a stirrer and a coil. Following this, the mixture was cooled to 90 °C and LpHera liquefaction enzyme preparation was added. The above temperature was maintained for 40 min, to ensure optimal starch dextrinization conditions. Subsequently, the temperature was reduced to 65 °C by passing cooling water through a coil, and the Saczyme saccharifying enzyme preparation was applied. In the case of the SHF technique, this temperature was maintained for the next 30 min, after which the mash was cooled to 30 °C. In the SSF method, however, the temperature was lowered to the set 30 °C immediately after the enzyme preparation was added.

In the case when potatoes were subjected to the PLS method, the next step following their cleaning was mechanical processing. The potato tubers were subjected to a mechanical process that involved the grinding of 15 kg of the potatoes to produce a pulp. The pulp was then placed into a tank, followed by addition of 2 L of tap water. The stirrer was turned on, and the mixture was heated using a water jacket that was heated by gas burners. As in the pressure-thermal method, when the pulp reached a temperature of 90 °C, the LpHera enzyme preparation, at the same dose, was added. The set temperature was maintained for 40 min, after which it was lowered to 65 °C, and Saczyme preparation was added. The hydrolysis and fermentation processes, using SHF and SSF techniques, were performed analogously to the previously described procedures for the raw material pretreated by the pressure-thermal method.

After cooling to a temperature of approximately 30 °C, the pH of the mashes was measured and adjusted to the level of 4.80 by the addition of a 25% *w*/*v* sulfuric acid (VI) solution. Subsequently, 3 L of sweet mash were transferred into fermentation flasks with a capacity of 6 L. The mashes were supplemented with the water solution of ammonium hydrogen phosphate, at a dose of 0.3 g/L of mash, to provide a source of nitrogen and phosphorus for yeast.

Potato mashes were fermented using dry distillery yeast strains. The yeast was initially hydrated and disinfected by incubating the cells suspended in a 25% *w*/*v* sulfuric acid solution at pH 2.0 for 15 min. The yeast cream was then added to the mash (without neutralisation) in the proportion: 0.3 g of dry yeast per 1 litre of mash. The inoculated mashes were carefully mixed prior to fermentation and placed in a thermostatic room at a temperature of 32 ± 1 °C. The flasks were closed with fermentation tubes filled with glycerine. In order to monitor the process, the flasks were mixed and weighed every 2 h. Once the weight of the flasks remained unchanged for an hour, the fermentation process was considered complete. The total fermentation time of the potato mashes ranged from 62 to 69 h.

### 3.3. Distillation of Alcohol from Fermented Mashes

The distillation of alcohol from the mashes after the completion of fermentation was conducted using the distillation unit, as previously described in our study [80]. The measurement of alcohol concentration in distillate streams was conducted by means of an Abbe refractometer. The distillation process was completed when the refractive index of the distillate stream approached 0.0%. The obtained distillates showed an ethanol concentration in the range of 14–20% *v*/*v*. In the next step, they were concentrated (without fractionation) to approximately 43 ± 1% (*v*/*v*) in a glass distillation apparatus equipped with a dephlegmator, according to Golodetz.

### 3.4. Analytical Methods

The moisture content of the potatoes used in this study was measured by drying the samples in the Radwag WPS-30S Moisture Analyser (RADWAG Balances and Scales, Radom, Poland) at 105 °C until a constant sample weight was reached. The total nitrogen was determined by the Kjeldahl method and calculated as protein (N × 6.25) [81].

Reducing sugars and total sugars (after acid hydrolysis of dextrins) were quantified by HPLC (1260 Infinity, Agilent Technologies, Santa Clara, CA, USA) with a refractometer detector (RID), following the methodology described by Dziekońska-Kubczak et al. [82]. The results were then converted to glucose equivalents using appropriate factors (maltose × 1.05; maltotriose × 1.07) and expressed as g glucose per 100 g of raw material. The starch content was calculated by determining the difference between the total and reducing sugars, applying a conversion factor of 0.9, and expressing the result as g per 100 g of raw material.

In potato sweet mashes, pH (HandyLab 750, SI Analytics GmbH, Mainz, Germany), extract content (refractometer, Atago, Tokyo, Japan), and concentrations of reducing sugars (maltotriose, maltose, glucose) and dextrins (HPLC) were determined. In the mashes after fermentation completion, the concentrations of ethanol, unfermented sugars, and fermentation by-products were quantified using the HPLC technique [82]. The fermentation efficiency was expressed as the ratio of the ethanol yield (obtained in the experiments) to the theoretical ethanol yield calculated using the ethanol fermentation equation.

The acidity of the potato distillates was determined using the titrimetric method, whereby the sample was diluted with an equal volume of CO_2_-free water and titrated with standard NaOH (0.1 mol/L) using phenolphthalein as an indicator. The results were expressed in g acetic acid/L alcohol 100% *v*/*v* [83].

The analysis of volatile compounds in the potato distillates was performed on an Agilent 7890 A gas chromatograph coupled to an Agilent MSD 5975 C mass spectrometer (Agilent Technologies, Santa Clara, CA, USA), according to the procedure given in the work of Pielech-Przybylska et al. [19]. A capillary column (Agilent VF-WAX MS; 60 m × 0.50 µm × 0.32 mm) was used to separate the compounds. The GC oven temperature was programmed to increase from 40 °C (maintained for 6 min) to 80 °C at a rate of 2 °C/min, then to 220 °C at 10 °C/min and maintained for 5 min. The flow rate of the carrier gas (helium) through the column was 2.0 mL/min. The injector temperature was 250 °C. The MS ion source, transfer line and quadrupole temperatures were 230 °C, 250 °C and 150 °C, respectively. The ionisation energy was 70 eV.

### 3.5. Statistical Analysis

All experiments and analyses were performed in duplicate (n = 2). The results were expressed as arithmetic means from the assays performed along with the standard deviations. Statistical analysis was performed in an R environment using RStudio version 2024.12.0 (RStudio Team, Boston, MA, USA) with multivariate analysis of variance (ANOVA) including interaction. Due to the limited number of repetitions, the assumption of a normal distribution was made, which should be treated as a limitation of the analysis. *p* values < 0.05 were considered to be statistically significant. Multiple comparisons using the Tukey method were used for post hoc comparisons. This method was chosen for its conservatism and ability to control family error, despite its lower statistical power compared to less restrictive methods. This approach reduces the risk of false positives, thereby increasing the reliability of the detected differences.

## 4. Conclusions

This study evaluated the impact of technological processes and selected yeast strains on the fermentation efficiency and quality of potato spirits.

It was demonstrated that yeast and, to a lesser extent, the method of raw material pretreatment were the factors determining the efficiency of potato mash fermentation. Furthermore, given that there were no significant differences in efficiency between separate hydrolysis and fermentation (SHF) and simultaneous saccharification and fermentation (SSF), the latter was considered the preferred solution. This approach reduces energy input, shortens the enzymatic hydrolysis time, and reduces the risk of microbial infections.

The volatile compound profile in potato distillates was found to be correlated to varying degrees with all the variables used during the technological process, which had a significant influence on the evaluation of the potato spirit samples. Equally importantly, all distillates produced met the requirements for potato spirit set out in European Union Regulation 2024/1143, despite elevated concentrations of certain volatile compounds, particularly acetaldehyde. The presence of these compounds resulted from the limited capabilities of the quarter-technical process, but it did not disqualify the final product in terms of compliance with EU law, since the criteria regarding the quality of the raw material, the production method, and the aromatic character of the potato spirit were maintained.

The research conducted has expanded our knowledge of potato distillate production technology. However, further experimental work is warranted, particularly with regard to improving the quality of potato distillate and the spirit drinks produced from it. The research results presented also complement the literature on distilling technology in the context of potato distillate production, in comparison to the more frequently discussed grain distillates.

## Figures and Tables

**Table 1:** Chemical composition of potato sweet mashes.

Method of Starch Liberation	System of Hydrolysis and Fermentation	Parameter	pH	Extract [°Blg]	Glucose [g/L]	Maltose [g/L]	Maltotriose [g/L]	Sum of Reducing Sugars, as Glucose [g/L]	Total Sugars, After Dextrin Hydrolysis as Glucose [g/L]	Dextrins [g/L]
Pressure-thermal (PT)	SHF	Mean	5.02 b	12.70 b	74.89 a	2.12 a	0.28 a	77.42 a	89.15 a	10.56 a
		SD	0.06	0.05	3.18	0.09	0.01	3.28	3.78	0.71
	SSF	Mean	4.82 b	12.40 b	75.70 a	2.04 a	0.24 a	78.11 a	85.04 ab	6.24 ab
		SD	0.04	0.07	3.21	0.09	0.01	3.31	3.51	0.26
Pressureless (PLS)	SHF	Mean	5.91 a	13.40 ab	77.22 a	1.06 b	0.01 b	78.35 a	79.87 b	1.38 c
		SD	0.04	0.06	3.28	0,04	0.00	3.32	3.38	0.06
	SSF	Mean	5.96 a	14.50 a	76.96 a	1.54 ab	0.05 b	78.64 a	88.26 a	8.67 ab
		SD	0.06	0.08	3.27	0.07	0.00	3.34	3.40	0.37

SHF—separate hydrolysis and fermentation; SSF—simultaneous saccharification and fermentation; SD—standard deviation; n.d.—not detected; mean values with different letters in the same columns differ significantly (multivariate analysis of variance, *p* < 0.05).

**Table 2:** Chemical characteristics of fermented potato mashes.

Method of Starch Liberation	System of Hydrolysis and Fermentation	Yeast	Parameter	Ethanol	Malto- Triose	Maltose	Glucose	Dextrins	Citric Acid	Succinic Acid	Formic Acid	Acetic Acid	Lactic Acid	Glycerol
				Concentration [g/L]										
Pressure-thermal (PT)	SHF	Ethanol Red	Mean	37.67 bc	0.271 a	0.059 bc	0.268 a	0.778 b	1.086 bc	0.424 b	0.722 bcd	1.723 bc	3.471 c	4.457 cd
			SD	0.16	0.003	0.020	0.009	0.172	0.032	0.020	0.039	0.070	0.810	0.108
		SaftSpirit	Mean	40.67 ab	0.183 b	0.066 bc	0.089 bc	0.104 d	2.061 ab	0.255 b	0.633 cd	0.463 d	n.d.	3.773 d
			SD	2.33	0.016	0.009	0.004	0.020	0.059	0.014	0.089	0.036	-	0.211
		Pinnacle	Mean	38.78 ab	0.198 b	0.065 bc	0.079 bcd	0.174 cd	2.232 a	0.236 b	0.583 cd	0.207 d	n.d.	3.359 d
			SD	3.03	0.009	0.007	0.007	0.008	0.157	0.011	0.069	0.054	-	0.221
	SSF	Ethanol Red	Mean	35.62 bc	0.042 cd	0.090 bc	0.043 cd	0.492 bcd	1.138 bc	0.582 ab	0.526 cd	3.059 a	3.994 bc	6.779 ab
			SD	0.79	0.011	0.012	0.017	0.211	0.240	0.373	0.460	0.920	0.672	1.523
		SaftSpirit	Mean	38.63 ab	0.019 cde	0.025 c	0.020 cd	0.495 bcd	0.798 c	0.338 b	0.290 d	1.119 cd	n.d.	4.575 cd
			SD	2.53	0.002	0.032	0.001	0.037	0.100	0.013	0.014	0.077	-	0.306
		Pinnacle	Mean	38.22 ab	0.023 cde	0.024 c	0.017 cd	0.574 bcd	1.142 bc	0.363 b	0.332 d	0.938 cd	n.d.	3.907 cd
			SD	1.45	0.006	0.029	0.001	0.203	0.317	0.007	0.021	0.140	-	0.286
Pressureless starch liberation (PLS)	SHF	Ethanol Red	Mean	33.03 c	0.010 de	0.083 bc	0.142 b	0.686 bcd	0.907 c	0.947 a	0.954 abcd	2.263 ab	5.567 a	6.851 ab
			SD	1.10	0.001	0.008	0.012	0.106	0.468	0.076	0.116	0.037	0.023	0.256
		SaftSpirit	Mean	39.93 ab	0.008 e	0.183 a	0.033 cd	0.700 bcd	1.081 bc	0.473 b	1.516 a	1.117 cd	0.608 d	5.932 abc
			SD	0.41	0.002	0.009	0.005	0.005	0.279	0.010	0.024	0.095	0.060	0.632
		Pinnacle	Mean	38.41 ab	0.011 de	0.185 a	0.050 cd	0.804 b	1.620 abc	0.338 b	1.044 abcd	0.512 d	n.d.	4.892 bcd
			SD	0.81	0.002	0.015	0.001	0.031	0.117	0.010	0.005	0.043	-	0.026
	SSF	Ethanol Red	Mean	35.65 bc	0.028 cde	0.198 a	0.336 a	0.251 bcd	0.653 c	0.939 a	1.479 ab	2.647 ab	4.952 ab	7.271 a
			SD	2.07	0.001	0.008	0.065	0.220	0.036	0.036	0.036	0.102	0.099	0.209
		SaftSpirit	Mean	42.85 a	0.027 cde	0.137 ab	0.008 de	0.701 bc	1.505 abc	0.359 b	1.302 abc	0.593 d	n.d.	5.216 bcd
			SD	0.33	0.001	0.009	0.004	0.053	0.007	0.016	0.425	0.036	-	0.034
		Pinnacle	Mean	42.81 a	0.045 c	0.091 bc	0.003 cd	1.659 a	1.111 bc	0.297 b	0.975 abcd	0.365 d	n.d.	4.820 bcd
			SD	1.07	0.017	0.042	0.003	0.299	0.523	0.015	0.197	0.171	-	0.272

SHF—separate hydrolysis and fermentation; SSF—simultaneous saccharification and fermentation; SD—standard deviation; n.d.—not detected; means in columns denoted by different letters are significantly different (multivariate analysis of variance, *p* < 0.05).

**Table 3:** Concentrations of carbonyl compounds and acetals in potato distillates.

Method of Starch Liberation	System of Hydrolysis and Fermentation	Yeast	Concentration	Acetone	Acet- Aldehyde	Diacetyl	Acetoin	Hexanal	Furfural	Decanal	Acetaldehyde Diethyl Acetal
				mg/L Alcohol 100% *v*/*v*							
Pressure- thermal (PT)	SHF	Ethanol Red	Mean	n.d.	94.71 bc	7.92 ab	n.d.	n.d.	9.59 abc	10.66 c	34.91 a
			SD	-	14.26	3.37	-	-	0.84	3.57	1.91
		SaftSpirit	Mean	4.15 d	230.55 ab	34.79 a	23.83 ab	n.d.	7.71 bc	8.80 c	68.90 a
			SD	0.39	53.17	17.00	12.79		0.72	0.17	12.40
		Pinnacle	Mean	3.43 d	117.24 bc	17.10 ab	5.77 ab	n.d.	7.87 bc	4.55 c	41.36 a
			SD	1.00	19.76	8.58	2.81		1.15	0.21	12.15
	SSF	Ethanol Red	Mean	5.13 cd	55.28 c	8.99 ab	n.d.	n.d.	10.86 ab	15.54 bc	19.19 a
			SD	0.75	13.00	3.54	-	-	4.60	5.42	3.98
		SaftSpirit	Mean	2.78 d	71.28 bc	15.68 ab	2.10 ab	n.d.	3.94 bc	6.89 c	22.47 a
			SD	0.25	11.85	2.94	1.60	-	0.23	2.33	4.60
		Pinnacle	Mean	4.15 d	151.00 abc	17.58 ab	12.70 ab	n.d.	4.16 bc	9.98 c	50.28 a
			SD	1.57	38.41	0.08	1.70	-	0.85	1.23	15.32
Pressureless starch liberation (PLS)	SHF	Ethanol Red	Mean	14.30 abc	227.96 ab	14.02 ab	84.14 a	1.02 a	4.72 bc	14.09 bc	72.04 a
			SD	1.30	46.33	12.82	66.92	1.44	1.13	3.63	12.92
		SaftSpirit	Mean	15.04 ab	84.68 bc	6.80 ab	5.96 ab	1.49 a	3.07 c	33.41 a	29.21 a
			SD	0.50	0.10	0.49	0.53	0.30	0.76	1.00	0.47
		Pinnacle	Mean	17.99 a	297.17 a	11.79 ab	11.51 ab	1.78 a	5.64 bc	27.62 ab	96.84 a
			SD	1.73	27.37	2.15	8.93	0.76	1.60	1.08	52.46
	SSF	Ethanol Red	Mean	21.46 a	133.05 abc	4.07 b	18.71 ab	1.78 a	15.38 a	34.45 a	48.15 a
			SD	7.38	78.65	0.93	20.29	0.75	0.12	9.09	32.61
		SaftSpirit	Mean	13.43 abc	168.31 abc	9.98 ab	10.42 ab	0.84 a	6.20 bc	12.92 c	55.66 a
			SD	0.87	4.14	3.37	1.19	0.69	0.83	0.88	2.26
		Pinnacle	Mean	8.70 bcd	209.96 abc	16.48 ab	14.79 ab	0.63 a	5.70 bc	12.88 c	63.53 a
			SD	0.95	88.19	9.67	2.90	0.16	2.71	1.02	24.41

SHF—separate hydrolysis and fermentation; SSF—simultaneous saccharification and fermentation; SD—standard deviation; n.d.—not detected; mean values with different letters in the same columns differ significantly (multivariate analysis of variance, *p* < 0.05).

**Table 4:** Concentrations of esters in potato distillates.

Method of Mash Preparation	System of Hydrolysis and Fermentation	Yeast	Concentration	Ethyl Acetate	Ethyl Propionate	3-Methylbutyl Acetate	Ethyl Hexanoate	Ethyl Octanoate	Ethyl Nonano- ate	Isobutyl Acetate	Ethyl Decanoate	Ethyl Formate
				mg/L Alcohol 100% *v*/*v*								
Pressure- thermal (PT)	SHF	Ethanol Red	Mean	60.47 f	0.32 bc	0.51 e	1.41 a	3.69 d	2.80 cd	2.43 a	5.67 bcd	1.47 bc
			SD	4.07	0.46	0.22	0.75	0.41	0.12	0.38	0.06	0.46
		SaftSpirit	Mean	81.69 cdef	1.92 a	1.76 de	1.57 a	3.61 d	n.d.	1.86 abcd	4.74 bcd	4.02 ab
			SD	5.69	0.24	0.59	0.39	0.11	-	0.34	2.05	2.38
		Pinnacle	Mean	76.5 def	2.16 a	1.36 de	1.30 a	3.51 d	n.d.	1.48 bcde	6.08 bcd	5.40 a
			SD	3.80	0.31	0.12	0.02	0.61	-	0.02	1.83	0.88
	SSF	Ethanol Red	Mean	134.18 abcde	n.d.	n.d.	1.52 a	3.78 d	1.07 de	1.60 bcde	4.70 bcd	6.18 a
			SD	45.1	-	-	0.26	0.47	1.52	0.08	1.64	1.02
		SaftSpirit	Mean	111.36 bcdef	1.35 abc	2.43 de	0.96 a	3.92 d	5.10 a	1.66 abcde	8.33 abc	4.44 ab
			SD	4.79	0.62	0.19	0.45	0.15	0.02	0.27	0.42	0.73
		Pinnacle	Mean	114.58 bcdef	1.52 ab	3.24 cd	1.22 a	3.89 d	4.84 ab	2.16 ab	9.21 ab	n.d.
			SD	15.86	0.72	0.53	0.05	0.41	0.29	0.14	1.23	-
Pressureless starch liberation (PLS)	SHF	Ethanol Red	Mean	91.83 cdef	n.d.	n.d.	1.27 a	10.86 a	1.18 cde	1.86 abcd	3.00 d	n.d.
			SD	3.87			0.31	1.23	0.51	0.01	0.25	-
		SaftSpirit	Mean	140.13 abcd	n.d.	5.12 bc	1.70 a	6.5 bc	2.49 cd	1.09 de	7.70 abcd	n.d.
			SD	4.52	-	0.64	0.24	0.17	0.09	0.08	0.16	-
		Pinnacle	Mean	169.09 ab	n.d.	5.75 ab	1.56 a	8.04 b	3.00 bc	1.32 cde	11.99 a	n.d.
			SD	31.53		1.83	0.19	1.26	0.01	0.09	2.66	-
	SSF	Ethanol Red	Mean	186.69 a	n.d.	0.56	0.62 a	7.08 bc	1.34 cde	1.36 cde	3.66 cd	n.d.
			SD	7.00	-	0.36	0.88	0.61	0.19	0.27	0.16	-
		SaftSpirit	Mean	69.16 ef	n.d.	2.07 de	0.97 a	5.51 cd	n.d.	0.97 e	6.46 bcd	n.d.
			SD	8.00	-	0.07	0.01	0.06	-	0.03	0.14	-
		Pinnacle	Mean	149.42 f	0.43 bc	8.00 a	2.05 a	8.26 b	n.d.	2.08 abc	12.42 a	n.d.
			SD	3.33	0.61	0.35	0.10	0.42	-	0.14	0.94	-

SHF—separate hydrolysis and fermentation; SSF—simultaneous saccharification and fermentation; SD—standard deviation; n.d.—not detected; mean values with different letters in the same columns differ significantly (multivariate analysis of variance, *p* < 0.05.

**Table 5:** Concentrations of methanol and higher alcohols in potato distillates.

Method of Starch Liberation	System of Hydrolysis and Fermentation	Yeast	Concentration	Methanol	1-Propanol	2-Methyl- 1-Propanol	1-Butanol	3-Methyl- 1-Butanol	2-Methyl- 1-Butanol
				mg/L Alcohol 100% *v*/*v*					
Pressure- thermal (PT)	SHF	Ethanol Red	Mean	1173.96 bc	547.66 e	829.62 cd	17.03 b	325.82 c	200.24 ef
			SD	36.30	37.60	18.40	1.10	17.80	8.09
		SaftSpirit	Mean	1096.88 cd	718.75 cd	902.34 c	13.8 b	510.96 b	316.07 cd
			SD	41.67	39.82	29.59	0.50	12.12	8.69
		Pinnacle	Mean	1076.89 cd	826.64 bcd	928.03 c	14.51 b	564.52 ab	310.38 cd
			SD	10.88	10.20	19.48	0.05	3.39	3.05
	SSF	Ethanol Red	Mean	1530.95 a	371.71 f	710.13 de	11.35 bcd	351.93 c	203.71 ef
			SD	138.99	93.07	58.74	5.89	15.75	0.39
		SaftSpirit	Mean	1389.01 ab	706.95 d	954.72 c	13.20 bc	524.3 b	327.15 bcd
			SD	97.60	46.43	23.66	1.13	4.19	6.52
		Pinnacle	Mean	1286.69 bc	870.90 b	1138.18 b	14.20 b	597.48 ab	343.84 bc
			SD	1.11	5.14	53.60	0.81	27.95	5.37
Pressureless starch liberation (PLS)	SHF	Ethanol Red	Mean	819.18 e	314.61 fg	632.33 ef	6.00 cd	321.5 c	166.20 f
			SD	63.43	26.45	35.19	1.09	20.13	11.49
		SaftSpirit	Mean	812.25 e	728.18 bcd	1255.6 b	10.53 bcd	639.71 a	415.80 a
			SD	26.52	17.28	20.24	1.17	12.21	13.62
		Pinnacle	Mean	820.34 e	1183.14 a	1436.99 a	27.03 a	545.11 ab	357.95 abc
			SD	55.60	13.04	18.99	0.35	73.02	50.93
	SSF	Ethanol Red	Mean	902.98 de	198.13 g	557.54 f	4.15 d	316.58 c	185.65 f
			SD	12.36	17.11	37.94	0.14	24.2	13.36
		SaftSpirit	Mean	742.06 e	422.74 ef	934.07 c	4.03 d	541.04 ab	385.87 ab
			SD	3.00	23.67	41.01	0.57	23.47	15.66
		Pinnacle	Mean	699.99 e	868.11 bc	965.38 c	15.62 b	530.02 b	263.05 de
			SD	40.96	36.98	44.33	0.38	9.38	0.59

SHF—separate hydrolysis and fermentation; SSF—simultaneous saccharification and fermentation; SD—standard deviation; n.d.—not detected; mean values with different letters in the same columns differ significantly (multivariate analysis of variance, *p* < 0.05).

**Table 6:** Mashes preparation design.

No	Method of Mash Preparation	System of Hydrolysis and Fermentation	Abbreviation of Variant
1	pressure-thermal (steaming)	separate hydrolysis/saccharification and fermentation (SHF)	PT-SHF
2	pressure-thermal (steaming)	simultaneous saccharification and fermentation (SSF)	PT-SSF
3	pressureless (PLS)	separate hydrolysis/saccharification and fermentation (SHF)	PLS-SHF
4	pressureless (PLS)	simultaneous saccharification and fermentation (SSF)	PLS-SSF

## Data Availability

The original contributions presented in this study are included in the article. Further inquiries can be directed to the corresponding author.

## Abbreviations

The following abbreviations are used in this manuscript:

PT	Pressure-thermal pretreatment
PLS	Pressureless starch liberation
SSF	Simultaneous Saccharification and fermentation
SHF	Separate hydrolysis and fermentation
